# Development and validation of the machine learning model for acute exacerbation of chronic obstructive pulmonary disease prediction based on inflammatory biomarkers

**DOI:** 10.3389/fmed.2025.1616712

**Published:** 2025-08-01

**Authors:** Ye Zhu, Meng Wang, Xin-Nan Gu, Cen Wang, Su-Min Deng

**Affiliations:** Department of Respiratory and Critical Care Medicine, Yixing People's Hospital, Yixing, Jiangsu Province, China

**Keywords:** chronic obstructive pulmonary disease, acute exacerbation, machine learning, inflammatory biomarkers, monocyte-to-lymphocyte ratio

## Abstract

**Objective:**

Acute exacerbation of chronic obstructive pulmonary disease (AECOPD) is a major cause of hospitalization and mortality in COPD patients. Current prediction methods rely primarily on clinical symptoms and physician experience, lacking objective and precise tools. This study aimed to integrate multiple inflammatory biomarkers to develop and compare machine learning models for predicting AECOPD, providing evidence for early intervention.

**Methods:**

This retrospective study included 763 COPD patients (443 AECOPD, 320 stable COPD), randomly divided into training (*n* = 534) and validation (*n* = 229) cohorts at a 7:3 ratio. Demographic characteristics, comorbidities, and inflammatory indices were collected, including neutrophil-to-lymphocyte ratio (NLR), platelet-to-lymphocyte ratio, monocyte-to-lymphocyte ratio (MLR), eosinophil-to-lymphocyte ratio (ELR), and basophil-to-lymphocyte ratio. After variable selection using least absolute shrinkage and selection operator (LASSO) regression, traditional logistic regression (LR) and three machine learning models—random forest, gradient boosting machine (GBM), and support vector machine—were constructed. Model performance was evaluated using receiver operating characteristic curves, calibration curves, and decision curve analysis, with SHapley Additive exPlanations (SHAP) analysis for feature importance interpretation.

**Results:**

The GBM model demonstrated superior performance with an area under the curve (AUC) of 0.900 (95%CI: 0.858–0.942), accuracy of 0.948, specificity of 0.952, and sensitivity of 0.944 in the validation cohort, significantly outperforming the traditional LR model (AUC = 0.870). SHAP analysis identified MLR (mean SHAP value = 0.5), NLR (0.35), and pulmonary heart disease (0.32) as the three most important predictive factors. AECOPD risk increased significantly with rising MLR and NLR values, while ELR showed a negative correlation with AECOPD risk. Decision curve analysis confirmed that the GBM model provided the highest net benefit within clinically relevant threshold ranges (0.2–0.8).

**Conclusion:**

The GBM model integrating multiple inflammatory indices effectively predicts AECOPD. Based on routine blood test indicators without requiring expensive additional tests, this model is particularly suitable for resource-limited primary healthcare settings, providing a precise tool for early identification and individualized treatment of AECOPD, potentially improving prognosis and quality of life for COPD patients.

## Introduction

Chronic obstructive pulmonary disease (COPD) represents a major global public health challenge, characterized by persistent airflow limitation and respiratory symptoms (1). The burden of COPD is particularly severe in China, among individuals aged 40 years and above, the overall prevalence rate is 13.7%, primarily attributed to prolonged exposure to tobacco smoke, environmental pollutants, and population aging (2, 3). Acute exacerbation of COPD (AECOPD), defined as a sudden worsening of respiratory symptoms (cough, sputum production, and dyspnea) beyond normal day-to-day variations with concomitant airflow limitation and systemic inflammation, constitutes the principal cause of hospitalization and mortality in COPD patients. Studies conducted in the Asia-Pacific region indicate in-hospital mortality rates ranging from 4 to 14% (4).

Accurate prediction of AECOPD holds significant clinical importance for reducing hospitalization rates, decreasing mortality, and improving patients' quality of life. Currently, AECOPD diagnosis predominantly relies on clinical manifestations and physician experience, lacking objective and precise predictive tools (5). Research indicates that over 50% of AECOPD episodes are triggered by bacterial and viral infections, resulting in enhanced airway and systemic inflammatory responses. Consequently, the identification of biomarkers reflecting inflammatory status is valuable for AECOPD prediction and early intervention (6).

Recent studies have demonstrated that inflammation indices derived from routine blood tests possess significant predictive value for AECOPD. Neutrophil-to-Lymphocyte Ratio (NLR), Platelet-to-Lymphocyte Ratio (PLR), and Monocyte-to-Lymphocyte Ratio (MLR) serve as reliable indicators of systemic inflammatory response and exhibit significant elevation in AECOPD patients compared to stable COPD patients, positively correlating with C-reactive protein (CRP) levels and disease severity (6, 7). Conversely, Eosinophil-to-Lymphocyte Ratio (ELR) demonstrates a reduction during acute exacerbations, exhibiting an inverse relationship with the aforementioned indicators (8, 9). These parameters, derived from standard hematological examinations, are economically viable and particularly suitable for implementation in primary healthcare facilities, providing accessible tools for early AECOPD identification.

With the advancement of machine learning technology, integrating these inflammatory biomarkers with clinical characteristics to construct predictive models has become feasible. Compared to traditional statistical methodologies, machine learning approaches can process complex non-linear relationships, integrate multidimensional data, and enhance predictive accuracy (10). Previous research has attempted to establish risk prediction models for respiratory failure in AECOPD patients or employ machine learning to identify patients at high risk for hospital readmission (11). However, comprehensive research utilizing multiple inflammatory biomarkers in conjunction with machine learning techniques for AECOPD prediction remains limited.

This study aims to develop and compare traditional logistic regression and various machine learning models for AECOPD prediction based on routine clinical data and inflammatory biomarkers (specifically NLR, PLR, MLR, and ELR). We will explore the predictive value of these inflammatory indices for AECOPD, evaluate the performance of different models, and determine the model with optimal clinical application value. Through this research, we anticipate providing more precise tools for early identification and intervention of AECOPD, optimizing stratified management and individualized treatment for COPD patients, thereby improving patient prognosis and quality of life.

## Materials and methods

### Study design and population

This was a retrospective, observational study approved by the Ethics Committee of Yixing People's Hospital. Informed consent was waived due to the observational nature of our retrospective analysis. We collected clinical data from patients with COPD hospitalized in our Respiratory Department from November 2023 to December 2024. Patients diagnosed with COPD according to the Global Initiative for Chronic Obstructive Lung Disease guidelines (12) were included. All COPD patients were diagnosed by pulmonologists based on smoking history, clinical symptoms, and spirometry measurements, with forced expiratory volume in 1 s to forced vital capacity ratio <70%.

Patients were categorized into AECOPD group and stable COPD group. AECOPD was defined as a sudden worsening of respiratory symptoms (cough, sputum production, and dyspnea) beyond normal day-to-day variations, accompanied by airway limitation and systemic inflammation, requiring hospitalization. Stable COPD was defined as COPD patients undergoing regular follow-up without acute exacerbation symptoms for at least 4 weeks.

Exclusion criteria included: (1) corticosteroid use prior to blood testing; (2) anti-infective medication use prior to blood testing; (3) hematological disorders; (4) other respiratory disorders; (5) mental or psychological disorders; (6) active pulmonary tuberculosis; (7) lung cancer or other malignancies; (8) severe hepatic or renal dysfunction; (9) autoimmune diseases; (10) recent surgical treatment; and (11) incomplete clinical data.

Eventually, 763 COPD patients were enrolled, including 443 AECOPD patients, 320 stable COPD patients, and 60 healthy controls. COPD patients were randomly divided into training (*n* = 534) and validation (*n* = 229) sets at a ratio of 7:3.

### Data collection

Demographic characteristics (age, gender, smoking status), comorbidities (hypertension, coronary heart disease, etc.), and laboratory test results were collected from all patients.

Blood samples were collected once at admission for baseline data analysis and between-group comparisons. Venous blood was drawn by professional nurses, using anticoagulant tubes for complete blood count analysis, which was performed within 2 h of collection. Another venous blood sample was collected simultaneously for serum separation to detect inflammatory markers including CRP. All blood tests were performed before any treatment to avoid potential influences on the results. The testing procedures were conducted according to standard laboratory protocols by professional technicians.

Laboratory parameters included complete blood count indices [white blood cell count, neutrophil count, lymphocyte count, monocyte count, eosinophil count, basophil count, platelet count, hemoglobin, red cell distribution width (RDW)], biochemical indicators, and inflammatory markers [N-terminal pro-brain natriuretic peptide (NT-proBNP), CRP]. Additionally, the following inflammatory indices were calculated: NLR, PLR, MLR, ELR, basophil-to-lymphocyte ratio (BLR), and systemic immune-inflammation index (SII, calculated as platelet × NLR).

### Model construction

Data preprocessing included normality testing for continuous variables and outlier identification and treatment. Outliers were identified using the IQR method and winsorized at the 5th and 95th percentiles. Missing values (<5%) were handled using multiple imputation by chained equations, creating 5 imputed datasets with sensitivity analyses performed.

Univariate and multivariate logistic regression analyses were conducted to identify risk factors associated with AECOPD and to construct a traditional logistic regression model. Least absolute shrinkage and selection operator (LASSO) regression was applied for feature selection to determine the optimal set of predictive variables. Hyperparameter tuning for machine learning models used grid search combined with 5-fold cross-validation, systematically optimizing key parameters including learning rate, tree depth, and minimum samples per leaf. Based on variables selected by LASSO regression, three machine learning models were developed: random forest (RF), gradient boosting machine (GBM), and support vector machine (SVM).

To assess model robustness and quantify overfitting, we performed supplementary analyses including bootstrap resampling, cross-validation, and optimism bias correction. Bootstrap resampling was conducted with 1,000 iterations from the original training set, with models tested on the validation set to calculate 95% confidence intervals for performance metrics. Five-fold cross-validation was implemented by randomly dividing training data into five subsets, using four for training and one for validation in each iteration, with mean values and standard deviations calculated across folds. Optimism bias correction was estimated using the bootstrap 0.632+ method to quantify the difference between training performance and true performance, with corrected performance calculated as: Corrected performance = Original performance – Optimism bias. Overfitting prevention strategies included cross-validation for model selection, LASSO regularization, early stopping criteria for gradient boosting models, maintaining reasonable events-per-variable ratios (>10:1), and learning curve analysis to monitor performance convergence.

### Statistical analysis

Statistical analyses were performed using SPSS 25.0 and R 4.1.0 software. Continuous variables were presented as mean ± standard deviation (x ± s) and compared using *t*-tests or Mann-Whitney *U*-tests; categorical variables were presented as numbers (percentages) and compared using χ^2^ tests. *P* < 0.05 was considered statistically significant. Model performance evaluation included area under the curve (AUC), accuracy, specificity, sensitivity, positive predictive value, negative predictive value, and F1 score. Calibration curves were used to assess model calibration performance, and decision curve analysis was employed to evaluate clinical utility. All models were constructed on the training set and validated on the validation set.

## Results

### Baseline characteristics

A total of 763 COPD patients were included in this study, with 534 (70%) in the training cohort and 229 (30%) in the validation cohort. The mean age of the patients was 71.5 ± 8.3 years, and 77.1% were male. There were 443 (58.1%) patients with AECOPD and 320 (41.9%) with stable COPD. No significant differences were observed in baseline characteristics between the training and validation cohorts (all *P*-values >0.05), indicating balanced grouping. The main comorbidities included pulmonary heart disease (37.0%), hypertension (29.0%), and coronary heart disease (28.0%). Hematological parameters and inflammatory markers for all patients are shown in Table 1.

**Table 1:** Baseline characteristics comparison between training and validation cohorts.

**Variables**	**Total**	**Training**	**Validation**	***P*-value**
Age (years)	71.5 ± 8.3	71.6 ± 8.4	71.3 ± 8.2	0.642
Gender (male)	588 (77.1%)	410 (76.8%)	178 (77.7%)	0.765
Smoking history	596 (78.1%)	415 (77.7%)	181 (79.0%)	0.685
Coronary heart disease	214 (28.0%)	148 (27.7%)	66 (28.8%)	0.742
Pulmonary heart disease	282 (37.0%)	195 (36.5%)	87 (38.0%)	0.695
Hypertension	221 (29.0%)	153 (28.7%)	68 (29.7%)	0.769
Diabetes	92 (12.1%)	63 (11.8%)	29 (12.7%)	0.725
Chronic kidney disease	23 (3.0%)	17 (3.2%)	6 (2.6%)	0.676
White blood cell count (× 10^9^/L)	8.42 ± 3.45	8.44 ± 3.46	8.37 ± 3.43	0.793
Neutrophil count (× 10^9^/L)	6.58 ± 3.17	6.62 ± 3.19	6.51 ± 3.13	0.649
Lymphocyte count (× 10^9^/L)	1.02 ± 0.57	1.01 ± 0.56	1.04 ± 0.58	0.516
Monocyte count (× 10^9^/L)	0.56 ± 0.31	0.57 ± 0.32	0.55 ± 0.30	0.414
Eosinophil count (× 10^9^/L)	0.11 ± 0.16	0.11 ± 0.16	0.11 ± 0.15	0.887
Basophil count (× 10^9^/L)	0.019 ± 0.015	0.019 ± 0.015	0.018 ± 0.015	0.320
Platelet count (× 10^9^/L)	242 ± 96	243 ± 97	240 ± 94	0.693
Hemoglobin (g/L)	128.4 ± 18.6	128.1 ± 18.8	129.1 ± 18.2	0.476
RDW (%)	14.3 ± 2.7	14.4 ± 2.8	14.2 ± 2.6	0.332
Albumin (g/L)	37.6 ± 4.8	37.5 ± 4.9	37.8 ± 4.7	0.412
NT-proBNP (pg/mL)	288 ± 1,110	288 ± 1,111	288 ± 1,109	0.995
NLR	7.86 ± 4.68	7.93 ± 4.72	7.70 ± 4.59	0.518
PLR	276 ± 182	279 ± 185	270 ± 176	0.524
MLR	0.66 ± 0.42	0.67 ± 0.43	0.64 ± 0.41	0.363
ELR	0.12 ± 0.13	0.12 ± 0.14	0.11 ± 0.12	0.289
BLR	0.022 ± 0.018	0.022 ± 0.019	0.021 ± 0.017	0.438
SII	1,874 ± 1,523	1,898 ± 1,546	1,821 ± 1,474	0.527
CRP (mg/L)	27.8 ± 39.5	28.2 ± 40.1	26.9 ± 38.2	0.673
AECOPD	443 (58.1%)	310 (58.1%)	133 (58.1%)	0.998
Stable COPD	320 (41.9%)	224 (41.9%)	96 (41.9%)	0.998

AECOPD, Acute Exacerbation of Chronic Obstructive Pulmonary Disease; RDW, Red blood cell distribution width; NT-proBNP, N-terminal pro-brain natriuretic peptide; NLR, Neutrophil to Lymphocyte Ratio; PLR, Platelet to Lymphocyte Ratio; MLR, Monocyte to Lymphocyte Ratio; ELR, Eosinophil to Lymphocyte Ratio; BLR, Basophil to Lymphocyte Ratio; SII, Systemic Immune-inflammation Index; CRP, C-reactive protein.

### Traditional logistic regression model

Factors associated with AECOPD were screened through univariate and multivariate logistic regression analyses (Table 2). Univariate analysis showed that gender, pulmonary heart disease, white blood cell count, neutrophil count, lymphocyte count, monocyte count, eosinophil count, basophil count, platelet count, hemoglobin, RDW, albumin, NT-proBNP, CRP, and inflammatory indices (NLR, PLR, MLR, ELR, BLR, SII) were significantly associated with AECOPD (*P* < 0.001). After multivariate analysis, gender (OR = 1.238, 95%CI: 1.156–1.326, *P* < 0.001), pulmonary heart disease (OR = 1.756, 95%CI: 1.249–2.468, *P* = 0.001), neutrophil count (OR = 1.234, 95%CI: 1.046–1.456, *P* = 0.013), lymphocyte count (OR = 0.560, 95%CI: 0.429–0.732, *P* < 0.001), RDW (OR = 1.352, 95%CI: 1.186–1.542, *P* < 0.001), NT-proBNP (OR = 1.425, 95%CI: 1.154–1.762, *P* = 0.001), CRP (OR = 1.362, 95%CI: 1.110–1.672, *P* = 0.003), NLR (OR = 1.642, 95%CI: 1.342–2.008, *P* < 0.001), PLR (OR = 1.328, 95%CI: 1.054–1.673, *P* = 0.016), MLR (OR = 2.695, 95%CI: 1.176–6.175, *P* = 0.019), ELR (OR = 0.147, 95%CI: 0.041–0.525, *P* = 0.003), BLR (OR = 0.053, 95%CI: 0.005–0.546, *P* = 0.014), and SII (OR = 1.482, 95%CI: 1.225–1.792, *P* < 0.001) were identified as independent predictors of AECOPD.

**Table 2:** Binary logistic regression analysis of factors associated with AECOPD.

**Variables**	**Univariate analysis**		***P*-value**	**Multivariate analysis**	***P*-value**
	**AECOPD (*****n*** **=** **310)**	**Stable COPD (*****n*** **=** **224)**		**Adjusted OR (95% CI)**	
Age (years)	235 (75.8%)	175 (78.1%)	0.532	-	-
Gender (male)	76.5 ± 7.8	70.8 ± 8.0	<0.001	1.238 (1.156–1.326)	<0.001
Smoking history	238 (76.8%)	177 (79.0%)	0.547	-	-
Coronary heart disease	83 (26.8%)	65 (29.0%)	0.573	-	-
Pulmonary heart disease	132 (42.6%)	63 (28.1%)	<0.001	1.756 (1.249–2.468)	0.001
Hypertension	86 (27.7%)	67 (29.9%)	0.586	-	-
Diabetes	42 (13.5%)	21 (9.4%)	0.139	-	-
Chronic kidney disease	11 (3.5%)	6 (2.7%)	0.580	-	-
White blood cell count (× 10^9^/L)	9.86 ± 3.42	6.44 ± 2.56	<0.001	1.158 (0.982–1.364)	0.082
Neutrophil count (× 10^9^/L)	8.23 ± 3.12	4.47 ± 2.07	<0.001	1.234 (1.046–1.456)	0.013
Lymphocyte count (× 10^9^/L)	0.82 ± 0.44	1.28 ± 0.57	<0.001	0.560 (0.429–0.732)	<0.001
Monocyte count (× 10^9^/L)	0.64 ± 0.33	0.47 ± 0.28	<0.001	0.714 (0.364–1.401)	0.328
Eosinophil count (× 10^9^/L)	0.08 ± 0.14	0.16 ± 0.17	<0.001	0.714 (0.364–1.401)	0.328
Basophil count (× 10^9^/L)	0.014 ± 0.012	0.026 ± 0.017	<0.001	0.752 (0.468–1.208)	0.241
Platelet count (× 10^9^/L)	258 ± 103	222 ± 85	<0.001	1.126 (0.895–1.418)	0.309
Hemoglobin (g/L)	121.3 ± 18.4	137.5 ± 15.2	<0.001	0.845 (0.681–1.047)	0.112
RDW (%)	15.6 ± 3.2	12.8 ± 1.6	<0.001	1.352 (1.186–1.542)	<0.001
Albumin (g/L)	35.1 ± 4.6	40.9 ± 3.3	<0.001	0.875 (0.736–1.039)	0.124
NT-proBNP (pg/ml)	354 ± 1,284	196 ± 865	<0.001	1.425 (1.154–1.762)	0.001
CRP (mg/L)	40.2 ± 46.3	11.5 ± 19.6	<0.001	1.362 (1.110–1.672)	0.003
NLR	11.65 ± 4.93	3.53 ± 1.63	<0.001	1.642 (1.342–2.008)	<0.001
PLR	354 ± 201	174 ± 95	<0.001	1.328 (1.054–1.673)	0.016
MLR	0.86 ± 0.46	0.39 ± 0.25	<0.001	2.695 (1.176–6.175)	0.019
ELR	0.07 ± 0.12	0.14 ± 0.15	<0.001	0.147 (0.041–0.525)	0.003
BLR	0.018 ± 0.018	0.026 ± 0.019	<0.001	0.053 (0.005–0.546)	0.014
SII	2,754 ± 1,686	768 ± 602	<0.001	1.482 (1.225–1.792)	<0.001

AECOPD, Acute Exacerbation of Chronic Obstructive Pulmonary Disease; RDW, Red blood cell distribution width; NT-proBNP, N-terminal pro-brain natriuretic peptide; NLR, Neutrophil to Lymphocyte Ratio; PLR, Platelet to Lymphocyte Ratio; MLR, Monocyte to Lymphocyte Ratio; ELR, Eosinophil to Lymphocyte Ratio; BLR, Basophil to Lymphocyte Ratio; SII, Systemic Immune-inflammation Index; CRP, C-reactive protein; OR, odds ratio; CI, confidence interval.

### Machine learning models

**Figure 1:**
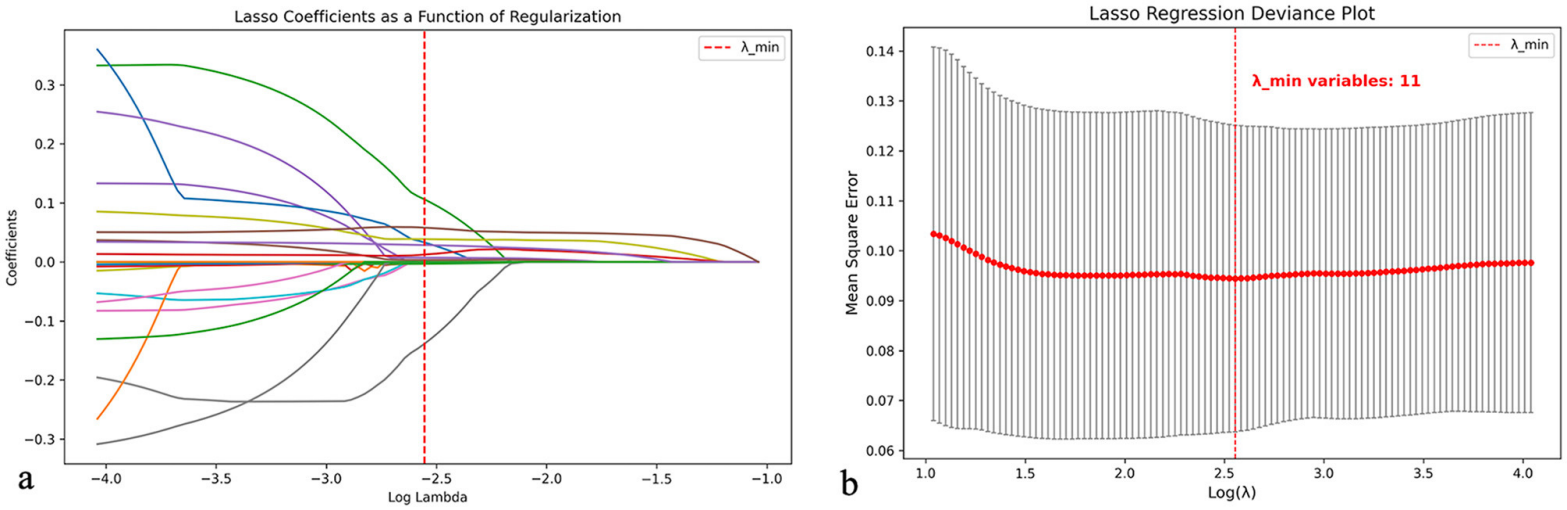
LASSO regression for feature selection: variable screening process for AECOPD prediction model. **(a)** LASSO coefficient paths showing how coefficient values change with increasing regularization (log λ). The vertical red dashed line represents the optimal λ value (λ_min = 0.00279) where the most relevant features are selected. **(b)** LASSO regression deviance plot displaying mean square error across different log(λ) values. At λ_min (vertical red dashed line), 11 key variables were selected for subsequent model construction. λ (Lambda): Regularization parameter that controls model complexity.

To optimize variable selection, the LASSO regression method was applied for feature screening. As shown in Figure 1, when the regularization parameter λ reached its minimum value (λ_min = 0.00279), 11 key variables were selected: MLR, NLR, pulmonary heart disease (PHD), PLR, CRP, ELR, age, NT-proBNP, BLR, RDW, and SII. Based on these variables, three machine learning models were constructed: RF, GBM, and SVM.

### Model performance comparison

The performance of the four models (traditional logistic regression and three machine learning models) in the training and validation cohorts is presented in Table 3. In the training cohort, the GBM model demonstrated the best performance with an AUC of 0.930 (95%CI: 0.891–0.969), accuracy of 0.948, specificity of 0.952, sensitivity of 0.944, positive predictive value of 0.953, negative predictive value of 0.942, and F1 score of 0.949. This was followed by the SVM model (AUC = 0.920, 95%CI: 0.881–0.959) and the RF model (AUC = 0.910, 95%CI: 0.871–0.949). The logistic regression model had an AUC of 0.870 (95%CI: 0.831–0.909). In the validation cohort, the GBM model maintained superior performance with an AUC of 0.900 (95%CI: 0.858–0.942), and other performance metrics also outperformed the other models.

**Table 3:** Performance of four models in training and validation cohorts.

**Model**	**AUC (95% CI)**	**Accuracy**	**Specificity**	**Sensitivity**	**PPV**	**NPV**	**F1 score**
**Training cohort**							
LR	0.870 (0.831–0.909)	0.805	0.793	0.817	0.811	0.799	0.814
GBM	0.930 (0.891–0.969)	0.948	0.952	0.944	0.953	0.942	0.949
RF	0.910 (0.871–0.949)	0.908	0.914	0.903	0.919	0.897	0.911
SVM	0.920 (0.881–0.959)	0.884	0.892	0.876	0.889	0.879	0.882
**Validation cohort**							
LR	0.850 (0.811–0.889)	0.803	0.826	0.778	0.800	0.806	0.789
GBM	0.900 (0.858–0.942)	0.882	0.909	0.852	0.893	0.873	0.872
RF	0.870 (0.831–0.909)	0.803	0.826	0.778	0.800	0.806	0.789
SVM	0.880 (0.841–0.919)	0.834	0.835	0.833	0.818	0.849	0.826

AUC, Area Under the Curve; CI, confidence interval; PPV, Positive Predictive Value; NPV, Negative Predictive Value; LR, Logistic Regression; RF, Random Forest; GBM, Gradient Boosting Machine; SVM, Support Vector Machine.

**Figure 2:**
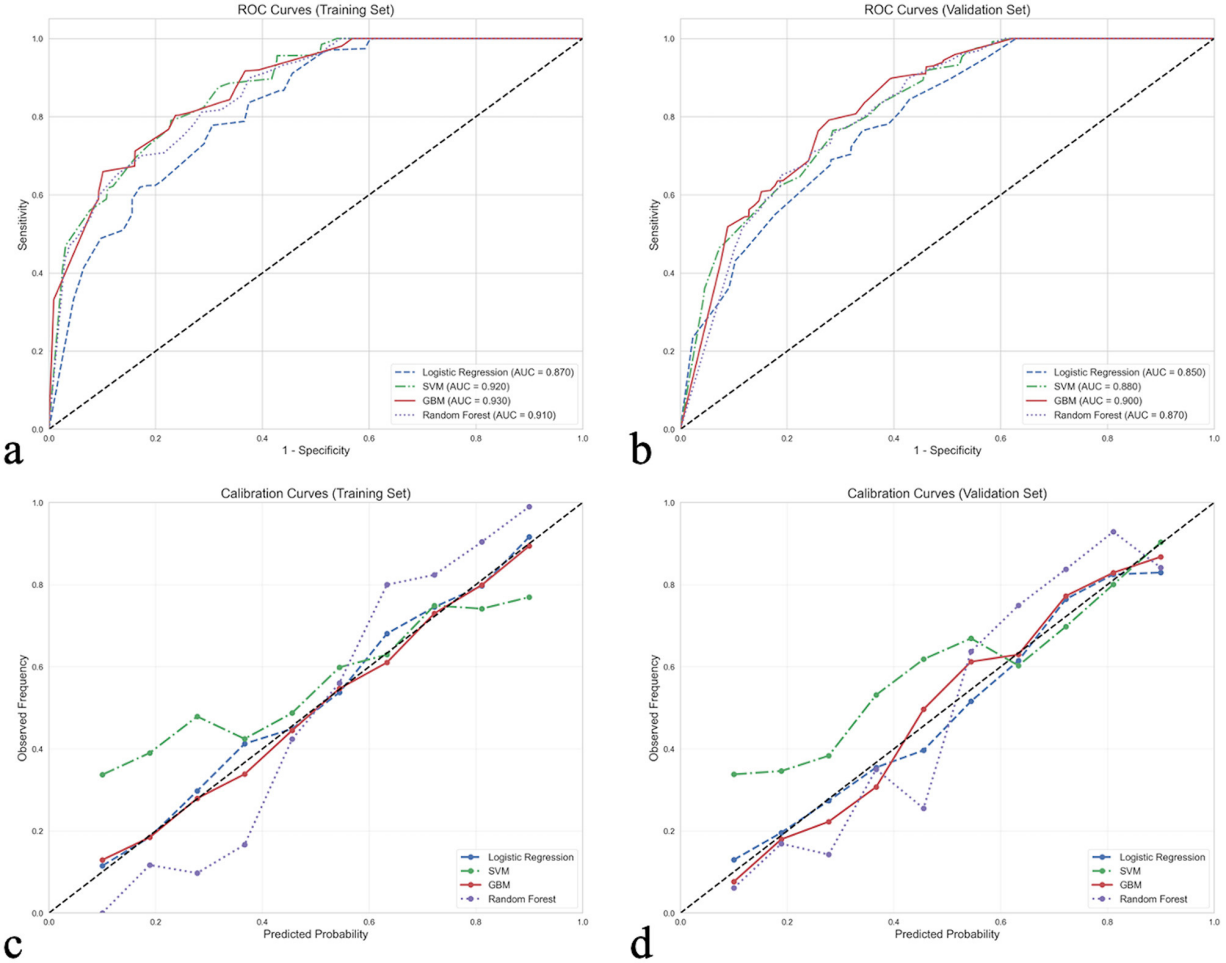
Performance comparison of four predictive models: discrimination ability comparison between machine learning and traditional statistical methods. **(a)** ROC curves for training set showing AUC values: GBM (0.930), SVM (0.920), RF (0.910), and LR (0.870). **(b)** ROC curves for validation set showing AUC values: GBM (0.900), SVM (0.880), RF (0.870), and logistic regression (0.850). **(c)** Calibration curves for training set comparing predicted probabilities with observed frequencies. **(d)** Calibration curves for validation set, with GBM maintaining the best calibration performance. Performance interpretation: AUC 0.5–0.7 (fair), 0.7–0.8 (good), 0.8–0.9 (excellent), >0.9 (outstanding); Perfect calibration: Predicted probability = actual probability, curve distributed along diagonal line.

Figure 2 displays the ROC curves (Figures 2a, b) and calibration curves (Figures 2c, d) for all four models in both the training and validation cohorts. The ROC curves indicate that all models had good discriminative ability, with the GBM model outperforming others in both cohorts. The calibration curves show that the GBM model's predicted probabilities aligned most closely with actual observed frequencies, indicating excellent calibration performance.

**Figure 3:**
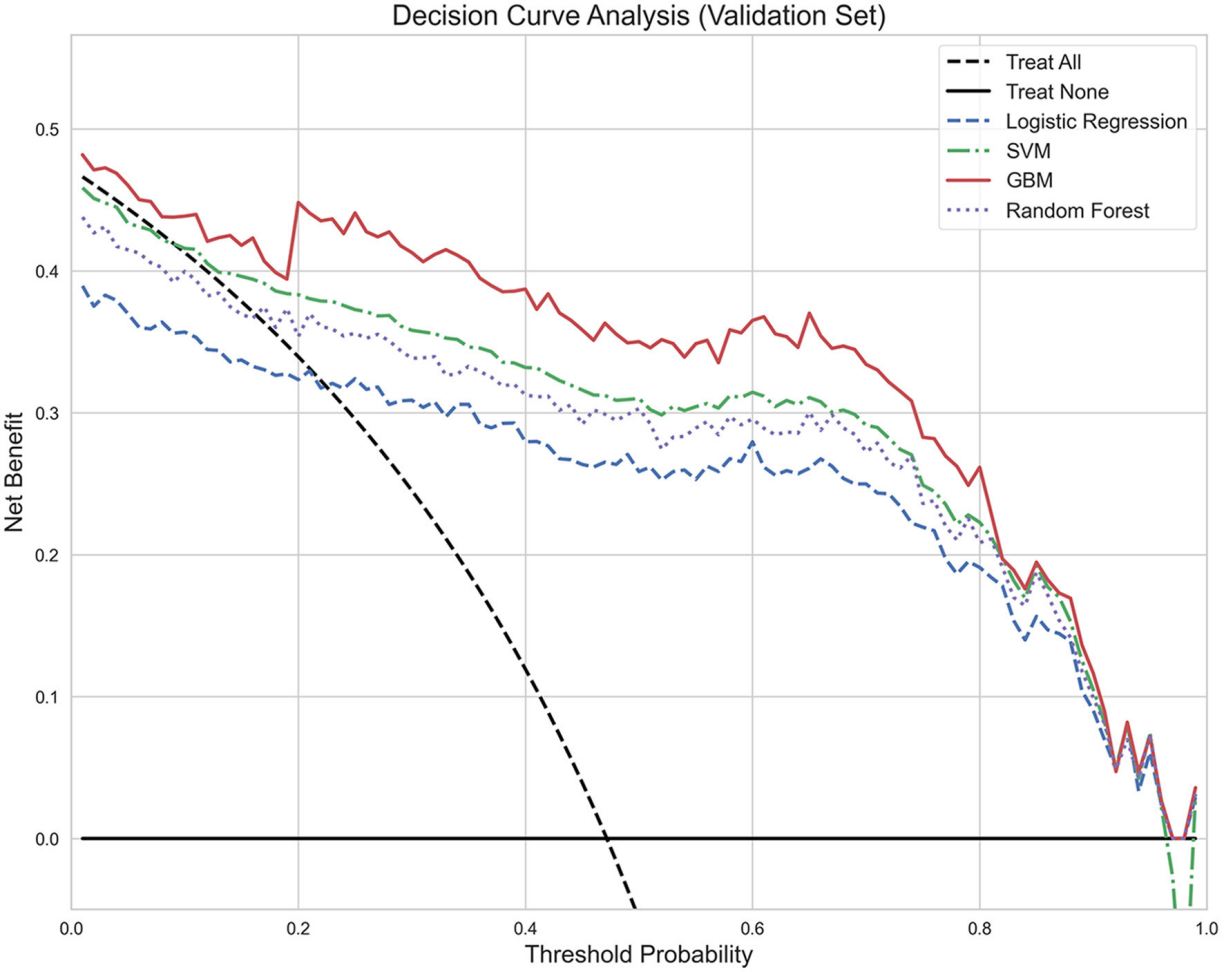
Decision curve analysis: clinical net benefit comparison of predictive models in validation set. The *y*-axis represents net benefit, while the *x*-axis shows threshold probability. The GBM model (red solid line) provides the highest net benefit across clinically relevant threshold probabilities (0.2–0.8) compared to SVM (green dash-dot line), RF (purple dotted line), LR (blue dashed line), and the reference strategies of “treat all” (black dashed line) and “treat none” (black solid line). Net Benefit: Clinical value of model-guided treatment at specific risk thresholds; Threshold Probability: Risk threshold for clinical decision-making; “Treat All”: Net benefit assuming all patients receive intervention; “Treat None”: Net benefit assuming no patients receive intervention; Interpretation: Model curves above reference lines indicate clinical application value.

To further evaluate the clinical utility of each model, decision curve analysis was performed (Figure 3). The results demonstrate that compared to “treat all patients,” “treat none,” and other models, the GBM model provided the highest net benefit across various threshold probabilities, especially within the clinically relevant threshold range of 0.2–0.8, further confirming the clinical application advantage of the GBM model.

### Model robustness validation results

Bootstrap resampling analysis based on 1,000 iterations demonstrated good statistical stability of GBM model performance on the validation set. The bootstrap 95% confidence intervals were: AUC 0.876–0.924, accuracy 0.864–0.901, sensitivity 0.825–0.878, and specificity 0.887–0.931. The relatively narrow confidence intervals indicated high reliability of model performance estimates.

Five-fold cross-validation results further validated model robustness. The GBM model achieved mean AUC of 0.895 ± 0.018, mean accuracy of 0.876 ± 0.022, mean sensitivity of 0.847 ± 0.026, and mean specificity of 0.902 ± 0.021 across the 5-fold. The small performance differences between folds, with all standard deviations <0.05, demonstrated consistent predictive performance under different data partitions.

Optimism bias correction analysis revealed an optimism bias of 0.012 for the GBM model, with a corrected AUC of 0.888, which was close to the original validation set AUC (0.900), suggesting low overfitting degree and good generalization capability.

### GBM model interpretation

**Figure 4:**
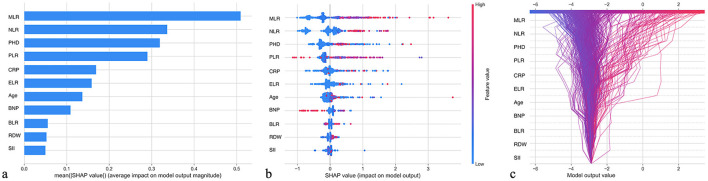
SHAP feature importance interpretation: contribution analysis of GBM model predictive factors. **(a)** Feature importance ranking based on mean SHAP values, with MLR (0.5), NLR (0.35), and PHD (0.32) identified as the top three predictors. **(b)** SHAP summary plot showing the impact of each feature on the model output, with red points indicating higher feature values and blue points indicating lower values. **(c)** SHAP waterfall plot demonstrating how increasing values of MLR, NLR, PLR, and CRP increase AECOPD risk prediction, while increasing ELR and BLR values decrease risk.

To enhance the interpretability of the GBM model, we employed the SHAP (SHapley Additive exPlanations) method to analyze feature importance and their impact on model predictions (Figure 4). The feature importance ranking in Figure 4a shows that MLR, NLR, and PHD were the three most important factors influencing model predictions, with mean SHAP values (average impact on model output magnitude) of 0.5, 0.35, and 0.32, respectively. Figure 4b illustrates the specific impact of each feature on model output, with red representing higher feature values and blue representing lower values. It can be observed that patients with high MLR, high NLR, and pulmonary heart disease generally had higher SHAP values, indicating that these factors were closely associated with increased AECOPD risk. Figure 4c demonstrates the relationships between feature values and model outputs through a waterfall plot, showing that as MLR, NLR, and other indicators increased, the predicted risk of AECOPD significantly increased; conversely, as ELR and BLR values increased, the predicted risk decreased, which is consistent with clinical understanding.

### Construction of AECOPD prediction nomogram based on GBM model

**Figure 5:**
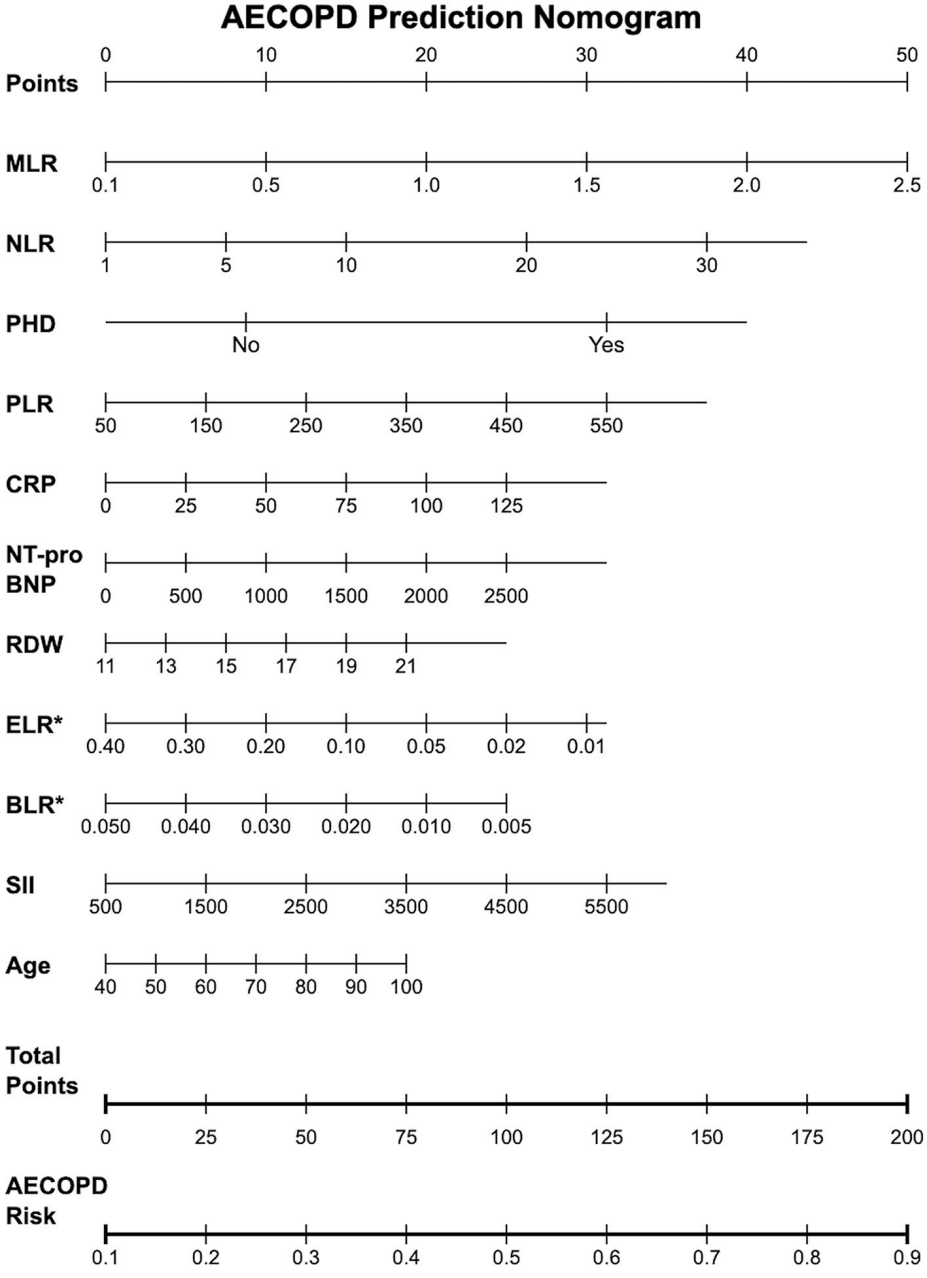
AECOPD prediction nomogram based on GBM model. This nomogram integrates 11 key predictive variables selected by LASSO regression.

To enhance the clinical practicality of our model, we constructed an AECOPD prediction nomogram based on the best-performing GBM model (Figure 5). This nomogram integrates the 11 key variables selected by LASSO regression, including MLR, NLR, PHD, PLR, CRP, NT-proBNP, RDW, ELR, BLR, SII, and age. The nomogram usage is simple and intuitive: clinicians locate the patient's actual measured values on the corresponding variable scales, then draw vertical lines upward to the Points scale to obtain corresponding points, sum all variable points to get the total points, and finally read the predicted risk probability on the AECOPD Risk scale based on the total points. This nomogram provides clinicians with a visual and easy-to-operate risk assessment tool, particularly suitable for routine clinical practice in primary healthcare institutions.

## Discussion

Despite being one of the main causes of death among COPD patients, the prediction and early identification of AECOPD still face significant challenges. Currently, clinical identification of AECOPD primarily relies on symptom assessment and physician judgment, lacking objective and accurate predictive tools. Several key limitations exist in current AECOPD prediction research: First, most studies focus on the predictive value of single biomarkers (such as CRP or procalcitonin), neglecting the potential advantages of integrating multiple inflammatory indicators. Although research has shown that indicators such as NLR and PLR are associated with AECOPD, comprehensive assessment of these indicators and their relative contribution weights remains limited (8, 13). Second, traditional statistical methods have limitations in predicting complex clinical outcomes like AECOPD, struggling to capture the non-linear relationships and variable interactions common in biological systems (14). Third, while some studies have attempted to construct risk prediction models for respiratory failure in AECOPD patients or identify high-risk patients for hospital readmission, these models are mostly based on single indicators or simple clinical features (11, 15). Finally, despite primary healthcare institutions bearing significant responsibility for COPD patient management, they often lack advanced equipment and technology, and existing models rarely consider practicality in resource-limited environments (16).

Addressing these limitations, our study integrated multiple inflammatory markers with clinical features using machine learning techniques to construct an efficient model for predicting AECOPD. We found that the GBM model performed best in the validation set (AUC = 0.900), significantly outperforming traditional logistic regression (AUC = 0.870). Through SHAP analysis, we identified MLR, NLR, and PHD as the three most critical factors for predicting AECOPD, consistent with previous research findings. Liao et al. (8) found that MLR is an independent predictor of in-hospital mortality in AECOPD patients (OR = 2.625) and increases with disease severity. Our results further quantify the relative contributions of different inflammatory indicators, providing more precise references for clinical decision-making. Notably, our study confirmed that ELR is negatively correlated with AECOPD risk, consistent with previous findings that ELR is significantly reduced in AECOPD patients and negatively correlated with CRP (4, 8). This finding supports the view that eosinophils may have a protective role in certain COPD phenotypes, providing new insights for precision medicine (17). Additionally, our research indicates that combining multiple inflammatory indicators such as NLR, PLR, MLR, and ELR can significantly improve predictive accuracy (11, 18–20).

From a pathophysiological perspective, our findings provide deeper mechanistic insights into AECOPD pathogenesis. MLR emerged as the predominant predictive factor, highlighting how activated monocytes orchestrate a dysregulated inflammatory response through enhanced pro-inflammatory cytokine production (interleukin-6, interleukin-8, tumor necrosis factor-alpha) and impaired phagocytic capacity during exacerbations (21, 22). The significant difference in MLR between AECOPD patients (0.86 ± 0.46) and stable COPD patients (0.39 ± 0.25) with a high odds ratio (OR = 2.695, 95% CI: 1.176–6.175) quantitatively captures this myeloid dysfunction. NLR, the second most influential factor, represents the critical imbalance between neutrophil-mediated tissue damage (via neutrophil extracellular traps formation, protease release, and delayed apoptosis) and compromised lymphocyte-regulated immunomodulation, reflecting both heightened innate immunity and suppressed adaptive responses (23, 24). PHD significance transcends comorbidity status, representing the pathophysiological consequence of pulmonary vascular remodeling and right ventricular dysfunction that fundamentally impairs cardiopulmonary reserve and ventilation-perfusion matching during exacerbations, aligning with Liao et al.'s (8) finding of PHD as an independent mortality predictor (OR = 2.324) through mechanisms of venous congestion and multiorgan dysfunction that synergistically aggravate respiratory failure.

Compared to existing AECOPD prediction methods, our study demonstrates significant advantages. Wang et al.'s (4) model based on serum biochemical markers achieved AUC values of 0.742–0.856, while our GBM model reached 0.900, representing a 5%−18% improvement. Unlike traditional BODE index and DECAF scores, our model integrates pathophysiology-driven inflammatory biomarkers without requiring expensive pulmonary function testing or imaging examinations. Methodologically, compared to Yin et al.'s (14) multivariable regression approach (AUC = 0.832), our machine learning method improved predictive performance by 8.2% through handling non-linear relationships and variable interactions. Importantly, SHAP interpretability analysis ensures clinical credibility of the model, which is lacking in most previous studies.

The GBM model developed in this study has significant clinical application prospects. First, the model is built on routine blood test indicators without requiring additional expensive tests, making it particularly suitable for resource-limited primary healthcare settings. This feature is especially important in China's advocated hierarchical diagnosis and treatment system for COPD, as primary and community hospitals often lack advanced equipment (25). Second, decision curve analysis shows that the GBM model has the highest net benefit within clinically relevant threshold ranges (0.2–0.8), indicating its significant practical application value. Third, SHAP analysis improves model interpretability, allowing clinicians to understand the key factors behind prediction results, enhancing model credibility. Finally, this model can effectively predict AECOPD risk, providing clinicians with an early intervention window, potentially reducing hospitalization and mortality rates. For example, high-risk patients (predicted probability >0.7) should receive immediate aggressive interventions including prioritized hospitalization, early bronchodilator and antibiotic therapy, consideration of systemic corticosteroids, and close respiratory function monitoring. Intermediate-risk patients (predicted probability 0.3–0.7) require enhanced monitoring with increased follow-up frequency within 24–48 h, optimization of existing treatment regimens, and individualized action plans for symptom deterioration. Low-risk patients (predicted probability <0.3) can maintain routine management with continued maintenance therapy and regular follow-up, focusing on disease education and lifestyle interventions.

To facilitate widespread adoption of this predictive model in clinical practice, we recommend implementing a standardized workflow. The proposed clinical implementation process includes: (1) routine blood testing upon patient admission to obtain baseline inflammatory markers before treatment initiation; (2) healthcare personnel using nomograms or integrated electronic tools to rapidly calculate AECOPD risk scores, typically requiring only 2–3 min; (3) developing individualized management plans based on risk stratification results, with low-risk patients receiving routine monitoring, intermediate-risk patients requiring enhanced observation frequency, and high-risk patients immediately initiating aggressive intervention measures; (4) dynamic monitoring of patient condition changes during hospitalization, reassessing risk levels when significant clinical status changes occur to guide treatment adjustments; and (5) systematic recording of patient clinical outcomes and treatment responses to provide data support for continuous model optimization and local calibration.

Clinical artificial intelligence implementation requires addressing key ethical considerations: ensuring algorithmic fairness without population bias; strict patient data privacy protection; maintaining decision transparency through SHAP analysis; obtaining informed consent; following regulatory validation requirements. Artificial intelligence should serve as decision support, with final clinical decisions remaining physician-directed.

This study has several noteworthy limitations that warrant consideration. First, the single-center retrospective design inherently introduces potential selection bias, limiting the generalizability of our findings across diverse patient populations and healthcare settings. To address this limitation, we plan multi-center external validation across five hospitals in different regions, targeting 2,000 COPD patients to ensure broader clinical applicability. Second, despite our comprehensive inclusion of multiple inflammatory indicators, we acknowledge the absence of other clinically relevant biomarkers—particularly procalcitonin—which might have provided additional discriminative value for infection-driven exacerbations. This limitation significantly impacts our model's ability to distinguish bacterial from viral or non-infectious exacerbations. Future model iterations should incorporate procalcitonin, presepsin, and other infection-specific biomarkers. Third, while our model effectively predicts AECOPD occurrence, it lacks the sophistication to differentiate between various etiological subtypes (bacterial, viral, or non-infectious), potentially obscuring important phenotypic distinctions that could guide targeted therapeutic approaches. Future work should develop specialized submodels incorporating pathogen-specific biomarkers to enable etiological classification and personalized treatment strategies. We propose integrating procalcitonin for bacterial infections, interferon-γ induced protein-10 for viral etiologies, and eosinophil indices for non-infectious triggers to reduce inappropriate antibiotic use and enable targeted therapy. Finally, our cross-sectional analysis captured inflammatory profiles at a single timepoint, failing to characterize the dynamic evolution of these biomarkers throughout the disease course and treatment response, which might offer valuable insights into recovery trajectories and therapeutic efficacy. As suggested by Mao et al. (25), more research is needed to validate the diagnostic and prognostic value of these indicators in AECOPD. Future research will focus on multi-center validation, clinical decision support system development, and evaluation of real-world patient outcomes.

In conclusion, the GBM model developed in this study can effectively integrate multiple inflammatory indicators to predict AECOPD with high accuracy and good clinical application prospects. The identification of MLR, NLR, and PHD as key predictive factors emphasizes the core role of systemic inflammation and cardiopulmonary function in AECOPD pathogenesis, providing a basis for developing tools to identify high-risk patients early. This facilitates timely intervention and individualized management strategies, ultimately improving prognosis and quality of life for COPD patients.

## Data Availability

The raw data supporting the conclusions of this article will be made available by the authors, without undue reservation.
